# Holocarboxylase Synthetase 1 Physically Interacts with Histone H3 in *Arabidopsis*

**DOI:** 10.1155/2013/983501

**Published:** 2013-02-12

**Authors:** Xi Chen, Hui-Hsien Chou, Eve Syrkin Wurtele

**Affiliations:** ^1^Department of Genetics, Development and Cell Biology, Iowa State University, Ames, IA 50011, USA; ^2^Department of Computer Science, Iowa State University, Ames, IA 50011, USA

## Abstract

Biotin is a water-soluble vitamin required by all organisms, but only synthesized by plants and some bacterial and fungal species. As a cofactor, biotin is responsible for carbon dioxide transfer in all biotin-dependent carboxylases, including acetyl-CoA carboxylase, methylcrotonyl-CoA carboxylase, and pyruvate carboxylase. Adding biotin to carboxylases is catalyzed by the enzyme holocarboxylase synthetase (HCS). Biotin is also involved in gene regulation, and there is some indication that histones can be biotinylated in humans. Histone proteins and most histone modifications are highly conserved among eukaryotes. HCS1 is the only functional biotin ligase in *Arabidopsis* and has a high homology with human HCS. Therefore, we hypothesized that HCS1 also biotinylates histone proteins in *Arabidopsis*. A comparison of the catalytic domain of HCS proteins was performed among eukaryotes, prokaryotes, and archaea, and this domain is highly conserved across the selected organisms. Biotinylated histones could not be identified *in vivo* by using avidin precipitation or two-dimensional gel analysis. However, HCS1 physically interacts with *Arabidopsis* histone H3 *in vitro*, indicating the possibility of the role of this enzyme in the regulation of gene expression.

## 1. Introduction

**Figure 1:**
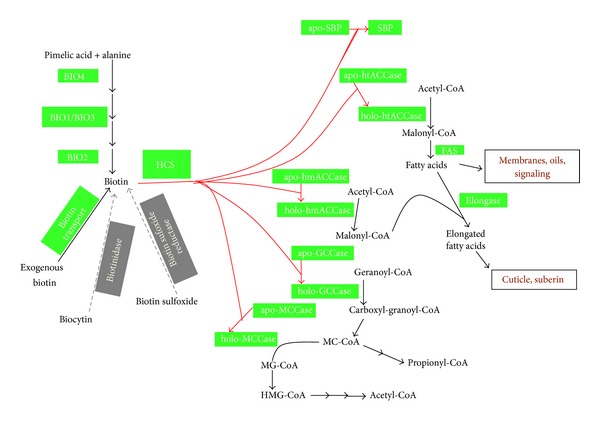
Biotin network in plants. Schematic map of the metabolism associated with biotin. Metabolites' names are in black text. Red arrows represent the biotinylating actions of HCS on biotin-dependent proteins. Black arrows represent other metabolic reactions characterized in plants. Enzymes identified by direct biochemical evidences are shaded in green boxes. Grey-dotted arrows represent the reactions characterized in humans or *E. coli* but not in plants so far. Enzymes not identified in plants but characterized in humans or *E. coli* are shaded in grey boxes.

Biotin is a water-soluble, B-complex vitamin that is required by all organisms [1]. The main role of biotin is to serve as a cofactor for carboxylases [2, 3]. Addition of biotin to carboxylases is catalyzed by holocarboxylase synthetase (HCS) in a two-step ATP-dependent reaction [4]. Based on the crystal structure of BirA, the *E. coli* HCS [3], the first step produces an intermediate biotinyl-5′-AMP (B-AMP). B-AMP is then transferred to a specific lysine residue of the carboxylase with the release of AMP [5]. Five biotin-dependent proteins have been characterized in plants [3]. One of them is a seed-specific protein SBP65 for biotin storage [6, 7]. The other four are all carboxylases: homomeric acetyl-CoA carboxylase, heteromeric acetyl-CoA carboxylase, geranoyl-CoA carboxylase, and methylcrotonyl-CoA carboxylase [8–11]. These enzymes are involved in many important metabolic pathways, such as gluconeogenesis, fatty acid synthesis, and amino acid catabolism [3] (Figure 1).

Biotin also participates in gene regulation [12–15], but the mechanisms are largely unknown. Evidence for histone biotinylation was reported in humans and this modification was attributed to HCS [16, 17]. Biotinylated histones were suggested to increase during mitotic DNA condensation, heterochromatin formation, gene silencing, and DNA repair [16, 18, 19]. These data indicated that biotin might be involved in human gene regulation by remodeling histones. Histones and most histone modifications are highly conserved [20]; thus, plants may use a similar mechanism involving biotin to regulate gene expression. HCS1 is the only functional enzyme in *Arabidopsis* for biotin ligation [21] and shares a conserved catalytic domain with humans. Therefore, we hypothesized that HCS1 also biotinylates histone proteins in plants. To test this hypothesis, we used two approaches: analysis of histone for modification by biotinylation *in vivo *and glutathione S-transferase (GST) pull-down assays to detect whether there was an interaction between HCS1 and *Arabidopsis* histone proteins *in vitro*. Biotinylated histones could not be identified *in vivo* by avidin precipitation or two-dimensional (2D) gel analysis. However, HCS1 pull-down assays indicate that HCS1 specifically binds to histone H3 protein *in vitro*. These results suggest that covalent modifications of histones by biotin may not naturally exist in *Arabidopsis*, although some types of physical interaction between HCS1 and histones may occur.

## 2. Results

### 2.1. The Functional Domain of HCS Proteins Is Highly Conserved

**Figure 2:**
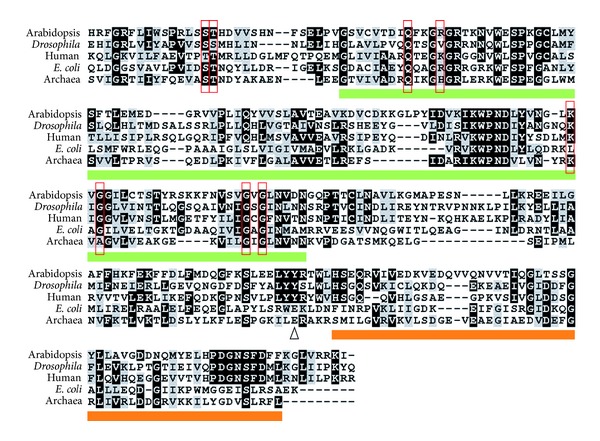
Multiple sequence alignments of HCS proteins. The highly conserved sequences of HCS proteins among different organisms were aligned using CLUSTALX [27]. Eight amino acids labeled by red boxes are required for biotin ligation according to the BirA crystal structure [22]. The BPL_lipA/B and BPL_C domains are underlined in green and orange, respectively. Δ is location of Y663 mutation in a patient with HCS deficiency.

HCS1 has two protein isoforms generated by alternative splicing: HCS1-T (containing a targeting signal for chloroplast and mitochondria localization) and HCS1-CY (not containing any targeting signal and accumulating in cytosol) [21]. HCS1-T and HCS1-CY share two protein domains: lipoate-protein ligase A/B (BPL_lipA/B) and biotin protein ligase C terminus (BPL_C). BPL_lipA/B is a catalytic domain for biotin ligation and was initially determined from the crystal structure of BirA [22]. This catalytic domain [4, 23] has a high identity (about 30%) across *Drosophila*, humans, *E. coli*, and *Pyrococcus abyssi* GE5 HCS proteins (Figure 2). Specifically, eight residues in or around the BPL_lipA/B domain are highly conserved across the selected HCS proteins (Figure 2). These eight residues directly contact biotin [22]. The BPL_C domain is thought to interact with ATP and the substrates [24]; it is also conserved across HCS proteins (Figure 2). In this domain, two motifs Leu-Tyr-Tyr-(Arg/Lys) and Pro-Asp-Gly-Asn-Ser-Phe-Asp have a high homology among eukaryotic organisms, but are not found in prokaryotes and archaea (Figure 2). The function of these two motifs is still unknown. A missense mutation in Leu-Tyr-Tyr-(Arg/Lys) was recently reported in a patient with human HCS deficiency [25]; thus, this motif may be important to HCS function. 

### 2.2. Biotinylated Histone H3 Could Not Be Identified in *Arabidopsis* Using Avidin Precipitation Analyses

**Figure 3:**
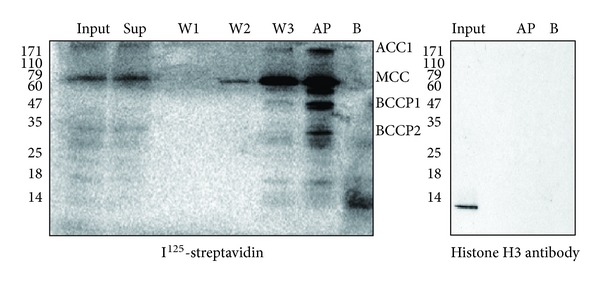
Avidin precipitation assays. The protein samples were analyzed by 15% SDS-PAGE. I^125^-streptavidin (left panel) and histone H3 antibody (right panel) were used in the western blots. Input: total protein extracted from *Arabidopsis*. B: blank control, where avidin beads were used only with release buffer. Sup: the supernatant after the avidin beads were incubated with *Arabidopsis* lysate overnight. W1/2/3: the supernatants for the first, second, and third washes separately after the incubation. AP: precipitation of total protein preformed with avidin beads. ACC1: homomeric acetyl-CoA carboxylase. MCC: methylcrotonyl-CoA carboxylase. BCCP1: biotin carboxyl carrier protein1, which is part of the heteromeric acetyl-CoA carboxylase. BCCP2: biotin carboxyl carrier protein2, which is part of the heteromeric acetyl-CoA carboxylase. Experiments were conducted in triplicate.

To investigate whether biotinylated histone H3 exists in *Arabidopsis*, total proteins were isolated from *Arabidopsis* and used for avidin precipitation. After avidin precipitation, proteins were separated by 15% SDS-PAGE and probed with I^125^-streptavidin and histone H3 antibody, respectively. The resultant western blots showed the biotinylated protein signals in the total protein extract from *Arabidopsis* (Figure 3, left panel, “Input” and “Sup” lanes) and highly accumulated signals in the avidin precipitate at the locations of biotinylated proteins [21] (Figure 3, left panel, “AP” lane). The negative control (avidin beads only) showed no positive signals (Figure 3, left panel, “B” lane). This result indicates that biotinylated proteins were precipitated successfully. However, when the same precipitates were probed with histone H3 antibody, no histone H3 proteins were detected. The result means that no biotinylated histone H3 proteins were precipitated by avidin beads (Figure 3, right panel, “AP” lane). Similar experiments were repeated under a wide variety of immunoprecipitation and western blot conditions, but none detected any biotinylated histone [26]. Taken together, the data suggest that biotinylated histone H3 could not be identified in an extended precipitation analysis, or the amount of biotinylated histone H3 is too low to be detectable [16].

### 2.3. Biotinylated Core-Histones Could Not Be Identified in *Arabidopsis* Using 2D Gel Analysis

**Figure 4:**
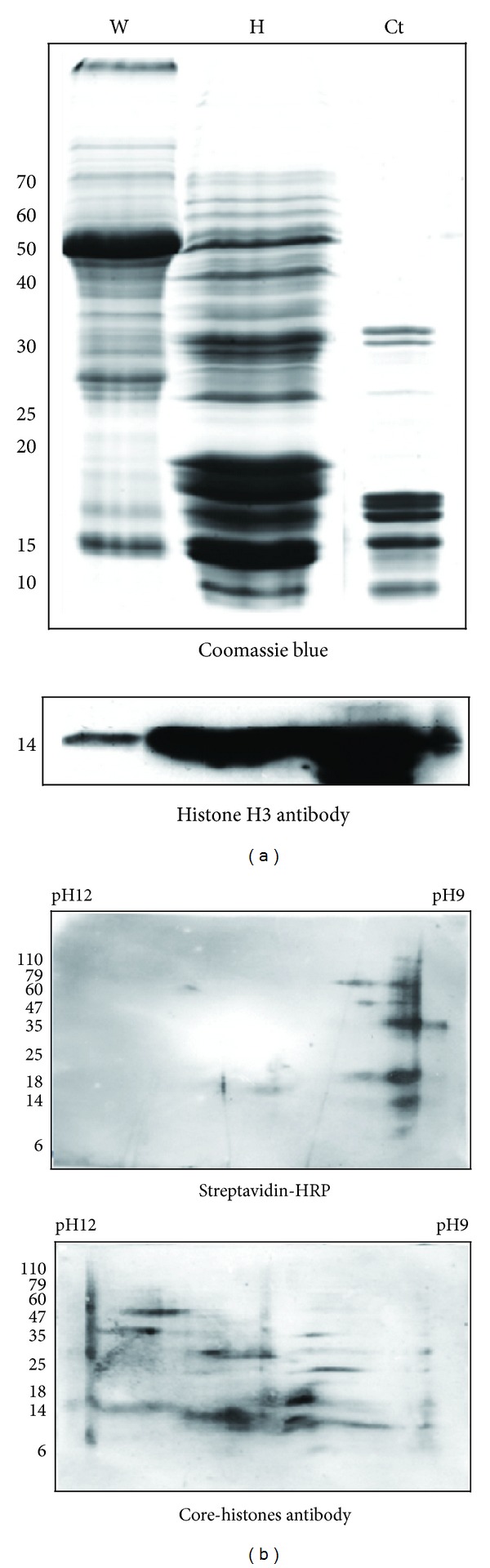
2D gel analysis. (a) Histone proteins were enriched from *Arabidopsis* young seedling lysate and stained with Coomassie Blue. The same amount of total and histone-enriched proteins was separated by 15% SDS-PAGE and the western blot results showed that histone proteins were enriched from *Arabidopsis* young seedling lysate. W: the total isolated proteins. H: the histone-enriched proteins. Ct: commercial calf thymus histones (Sigma). (b) The histone-enriched proteins were analyzed by 20% SDS-PAGE with pH gradient from 9 to 12. The biotin signals that concentrate at pH 9 versus the core-histones signals that distribute between pH 10 to 11. Experiments were conducted in triplicate.

2D gel analysis was used to investigate whether any core-histones (H2A, H2B, H3, and H4) are biotinylated in *Arabidopsis*. Histone-enriched proteins were isolated from *Arabidopsis* by sulfuric acid precipitation. Compared to the same amount of total protein extracted by using neutral lysis buffer (Figure 4(a), “W” lane), histone-enriched proteins were highly accumulated by using acid buffer (Figure 4(a), “H” lane). Commercial calf thymus histones were used as control (Figure 4(a), “Ct” lane). The histone-enriched proteins were subjected to isoelectric focusing using a gradient of pH 9–12 and separated in a second-dimension by 20% SDS-PAGE. Biotin signals were detected by streptavidin-HRP (Figure 4(b)); they are mainly distributed on the right side (~pH 9), where the known *Arabidopsis* biotinylated proteins (biotin-dependent carboxylases) are located (all these enzymes are neutral proteins). In contrast, when probed for the location of histone proteins using antiserum to core-histones, most signals are concentrated in the central area (~pH 10 to 11), where histones, all basic proteins, are located (Figure 4(b)). No overlap in locations is detectable between the signals from these two antibodies. This suggests that no core-histone proteins are biotinylated in *Arabidopsis*.

### 2.4. HCS1 Interacts with *Arabidopsis* Histone H3 Directly *In Vitro*

**Figure 5:**
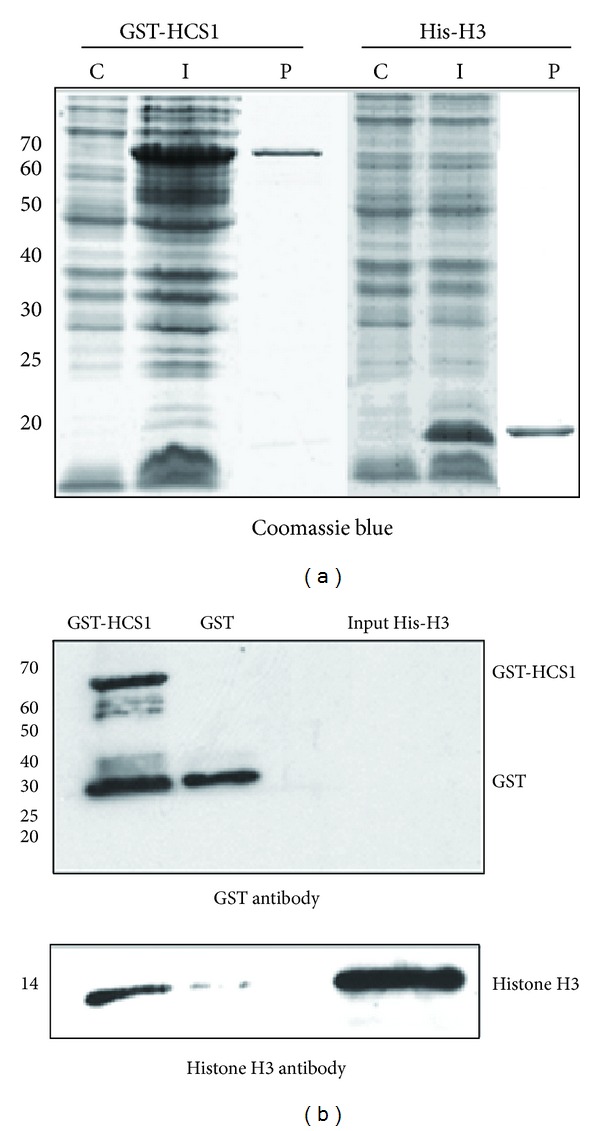
HCS1 interacts with *Arabidopsis* histone H3 directly *in vitro*. *E. coli* lines that contain GST-HCS1, His-H3, or GST construct were induced to express these genes. After induction, total protein was extracted from the cell lines. Recombinant His-histone H3 protein was purified from *E. coli* by using metal affinity chromatography. Recombinant GST-HCS1 or GST alone was purified from *E. coli* by using glutathione-sepharose beads. Proteins were subjected to GST pull-down assays with GST-HCS1 or GST alone and probed by western blot to detect whether there was an interaction between HCS1 and histone H3. (a) Proteins were analyzed by 15% SDS-PAGE and stained with Coomassie Blue. I: total proteins extracted from *E. coli* containing a GST-HCS1 or a His-H3 construct. C: total proteins extracted from control *E. coli* (not containing any vector). P: recombinant GST-HCS1 or recombinant His-H3 proteins. (b) Western blot of proteins after GST pull-down to detect whether there was an interaction between HCS1 and histone H3. GST-HSC1 fusion protein and GST were detected with GST antibody; Histone H3 was detected with corresponding antibody. GST-HCS1: the proteins released from the beads coupled with GST-HCS1 after being incubated with His-histone H3 proteins; Histone H3 pulled down by GST-HCS1 was detected. GST: the proteins released from the beads coupled with GST control after being incubated with His-histone H3 proteins; no histone H3 was detected. Input His-H3: 10% of the total input recombinant His-histone H3 proteins; Histone H3 was strongly detected as expected. Experiments were conducted in triplicate.

To identify whether HCS1 physically interacts with histone proteins, a GST pull-down assay was performed. Total RNA was prepared from *Arabidopsis* whole seedlings, and the full-length *histone H3* and *HCS1* RNA sequences were obtained by amplification of *histone H3* (AT1G09200) and *HCS1* (AT2G25710) with transcript-specific primers. The full-length *HCS1* cDNA was cloned into pGEX4T vector (GST-tagged) and the full-length cDNA of *Arabidopsis histone H3* was cloned into pDEST17 vector (His-tagged). After transforming each vector into *E. coli*, GST-HCS1 and His-histone H3 protein expressions were induced by isopropyl **β**-*d*-thiogalactoside (IPTG) or L-arabinose (Figure 5(a)).

A GST pull-down assay was used, in which recombinant proteins were purified from *E. coli* using glutathione beads or metal affinity chromatography. In this assay, purified GST-HCS1 proteins were incubated with glutathione-sepharose beads for 1 hr followed by addition of His-Histone H3 protein. After overnight incubation at 4°C, beads were washed extensively with assay buffer (10 mM Hepes, 100 mM NaCl, and 10 mM *β*-mercaptoethanol, pH 7.0) and resuspended in 2X SDS loading buffer. After boiling and centrifugation, the supernatant was separated by 15% SDS-PAGE. The proteins in each sample were separated by 15% SDS-PAGE and transferred to a membrane. The membrane was cut into two, such that the top portion contained proteins from ~20 kD to ~110 kD and the bottom portion contained proteins from ~6 kD to ~20 kD. The top portion was probed with GST antibody to detect any GST or the GST-fusion protein GST-HCS1; the bottom portion was probed with histone H3 antibody. The resultant western blots revealed that His-histone H3 proteins are pulled down by GST-HCS1 fusion proteins, but not by a GST protein control (Figure 5(b)), indicating that HCS1 specifically interacts with *Arabidopsis* histone H3 *in vitro*.

## 3. Discussion

Steven Stanley et al. first discovered that humans may have histones modified by biotin [16]. Biotinylated sites were identified on H2A (lysines 9, 13, 125, 127, and 129), H3 (lysines 4, 9, and 18) and H4 (lysines 8 and 12) [28–31]. As with other histone modifications, the levels of biotinylation of histones change during a variety of cellular processes [16, 18, 19]. Histones have been reported to be biotinylated by human HCS, human biotinidase, and *E. coli *BirA *in vitro* [17, 29, 32, 33], but only by human HCS *in vivo* [17]. Biotinidase is an enzyme required for recycling biotin from biocytin (lysine-biotin complex), which is essential for animals, but has not been identified in plants [34]. In *Arabidopsis*, HCS1 is the only known candidate for a functional biotin ligase that might biotinylate histones.

Protein alignments show that BPL_lipA/B, the catalytic domain of HCS proteins, is highly conserved across *Arabidopsis*, *Drosophila*, human, *E. coli*, and *Pyrococcus abyssi* GE5. In the canonical reaction, BPL_lipA/B catalyzes biotinylation on a specific lysine residue of each biotin-dependent carboxylase [1, 29]. This lysine residue locates within a highly conserved Ala-Met-Lys-Met motif [29], and each biotin-dependent carboxylase possesses only one specific lysine residue for biotinylation. Conversely, in histones, the biotinylated lysine residues are not found in a consistent region, and a histone protein usually has multiple biotinylated sites [28–31]. This suggests that a novel mechanism may be used when HCS biotinylates histones. *In vitro* experiments indicate that human HCS or *E. coli* BirA catalyzes biotin onto AMP to form B-AMP. Surprisingly, B-AMP can be attached to recombinant human H2A automatically without the need of enzymes [31]. Due to the high homology in the HCS catalytic domain among *Arabidopsis*, humans, and *E. coli*, we hypothesize that HCS1 may also biotinylate *Arabidopsis* histone H3 *in vitro* automatically. Our studies indicate that HCS1 indeed interacts with *Arabidopsis* histone H3 *in vitro* (Figure 4), partly validating our hypothesis. 

Immunofluorescence studies showed that the majority of human HCS localizes to the nucleus rather than the cytosol; thus, if it acts on histones in humans, it would likely act *in situ* in the nucleus [17]. *Arabidopsis* HCS1 has two protein isoforms: HCS1-T and HCS1-CY [21]. HCS1-T processes a dual targeting signal for chloroplast and mitochondria localization, whereas HCS1-CY only accumulates in cytosol without any characterized targeting signal [21]. Therefore, if any interaction between HCS/biotin and histones occurs in *Arabidopsis*, the modification would likely occur in the cytosol, before export of histone into the nucleus. 

The notion that humans possess biotinylated histones has been questioned recently [35, 36]. Three independent methods, [^3^H]-biotin uptake, western blot, and mass spectrometry analysis, were used to recheck the occurrence of human histone biotinylation [35, 36]. Using each method, biotinylated histones could be identified from *in vitro* experiments, but were not detected in any *in vivo* experiment. Even the research group members that originally reported biotinylated histones doubted their own results, finding that the avidin-HRP probe they used for validating biotinylated histones *in vivo* may not be credible [35]. Researchers also tested whether BirA can biotinylate recombinant human histone H2A in *E. coli* by increasing the amounts of radioactive-labeled biotin that was supplied to the medium. Although the radioactive signal from p-67 (a biotin-acceptor protein used as control) was easily detected, no biotinylated recombinant H2A was identified (unpublished data). These studies suggest that caution should be exercised in detection of biotinylated histones. 

Thus, to date it has been challenging to detect biotinylated histones isolated from either humans [35] or *Arabidopsis* (in this study). However, HCS1 itself does interact with *Arabidopsis* histone H3 *in vitro*. If HCS1/biotin interacts with histones in *Arabidopsis in vivo*, the interaction may involve a mechanism different from the covalent biotinylation that occurs to carboxylases. Understanding such an interaction may provide insight as to how biotin regulates gene expression rather than through biotinylated histones.

## 4. Material and Methods

### 4.1. Plant Materials

*Arabidopsis* seeds were surface-sterilized and dispensed into solid MS medium. Solid MS medium in Petri dishes contains 4.3 g/L MS (Murashige and Skoog salts), 1X B5 vitamins (100 *μ*g/mL myoinositol, 1 *μ*g/mL pyridoxine hydrochloride, 1 *μ*g/mL nicotinic acid and 10 *μ*g/mL thiamine hydrochloride), and 0.6% (w/v) agar gel, buffered with 2.56 mM MES at pH 5.7 [37]. After two-week growth, *Arabidopsis* whole seedlings were collected for RNA preparation and histone-enriched protein extraction.

### 4.2. Cloning of Full-Length Histone H3 and HCS1 Proteins

Total RNA was prepared from *Arabidopsis* whole seedlings. After TRIzol (Invitrogen) extraction, first-strand cDNAs were produced by Superscript II reverse transcriptase (Invitrogen) with oligo dT primer according to the manufacturer's instruction. PCR fragments corresponding to *histone H3* (AT1G09200) and *HCS1 *(AT2G25710) were amplified with the primers listed in Table 1.

### 4.3. Overexpression of Recombinant His-Histone H3 and GST-HCS1 Proteins

Amplified PCR fragments for *histone H3* were cloned first into an entry vector pENTR/SD/D-TOPO, which was then recombined with a binary vector pDEST17 [38]. The construct of His-histone H3 was verified by sequencing and was transformed into *E. coli* BL21 (AI) strains (Invitrogen). After 4 hr induction by addition of L-arabinose to the *E. coli* culture media, total proteins were separated by 15% SDS-PAGE [39].

Amplified PCR fragments for *HCS1* were digested with EcoRI and XhoI. They were then ligated into the corresponding digested sites on the pGEX4T vector. The construct of GST-HCS1 was verified by sequencing and was transformed into *E. coli* BL21 (DE3) strains. After 4 hr induction by addition of IPTG to the *E. coli* culture media, total proteins were separated by 15% SDS-PAGE [39]. 

### 4.4. Purification of Recombinant His-Histone H3 and GST-HCS1 Proteins

*E. coli* cells BL21 (AI or DE3) were grown in 1 L flasks to an absorbance of OD 0.5. Then, cells were pelleted, washed, and lysed with lysis buffer (lysozyme 100 mg/L, 10 mM DTT, 1 mM PMSF, and 1% Triton in PBS buffer) [40, 41]. Crude lysate of GST-HCS1 was coupled to glutathione-sepharose beads (Sigma). After overnight incubation at 4°C, the beads were washed extensively with PBS buffer (137 mM NaCl, 2.7 mM KCl, 10 mM sodium phosphate dibasic, and 2 mM potassium phosphate monobasic, pH 7.5). Recombinant GST-HCS1 proteins were released with elution buffer (10 mM Tris, 150 mM NaCl, and 20 mM reduced glutathione, pH 7.5) and separated by 15% SDS-PAGE. Recombinant His-histone H3 proteins were purified with Ni-NTA beads (Novagen) by following the manufacturer's instruction and separated by 15% SDS-PAGE. 

### 4.5. Avidin Precipitation Assays

*Arabidopsis* whole seedlings were homogenized in extraction buffer (20 mM pH 8.0 Tris-HCl, 10 mM EDTA, 1 mM EGTA, 150 mM NaCl, 2 mM Na_3_VO_4_, 0.2% Triton X-100, 0.2% NP-40, and 1 mM phenylmethylsulfonyl fluoride). The mixture solution was sonicated three times and then centrifuged at 16,000 g for 10 minutes at 4°C. 250 *μ*L supernatant was incubated with the NeutrAvidin beads (Thermo Scientific) which had been washed before two times with wash buffer A (1% NP-40, 0.1% SDS in PBS) and two times with PBS [42]. After each incubation or washing step the resin was pelleted by centrifugation at 100 g for 30 s. After overnight incubation at 4°C, the supernatant was removed and the resin was washed one time with wash buffer A one additional time with wash buffer B (0.4 M NaCl in wash buffer A) and one final time with 50 mM Tris-HCl, pH 7.5. To elute biotinylated proteins, 50 *μ*L release buffer (2% SDS, 30 mM biotin, 50 mM phosphate, 100 mM NaCl, 6 mM urea, 2 M thiourea) were added to the resin, which was then incubated for 15 min at RT and another 15 min at 96°C. Afterwards, the resin was pelleted by centrifugation for 5 min at 16,000 g and the resulting supernatant was collected and separated by 15% SDS-PAGE. When the membrane was probed by primary antibody of histone H3 (Iowa State University), the antibody was diluted 1 : 500 and the secondary antibody was diluted 1 : 5,000. Signal bands were detected with the Western Lightning chemiluminescence reagent (Perkin Elmer Inc.) After the membrane was probed by I^125^-streptavidin (8.0 × 10^5^ cpm), the radiolabeled signal was subsequently detected using the Typhoon scanner (GE).

### 4.6. Preparation of Histone-Enriched Proteins from *Arabidopsis* Whole Seedlings

After two-week growth, *Arabidopsis* whole seedlings were excised and homogenized in homogenization buffer (1 M pH 9.5 Tris-HCl, 0.5 M pH 8.0 EDTA, 1 M KCl, 1 M sucrose, 0.1% (v/v) 2-mercaptoethanol, and 1 M spermine) [43]. The homogenized solution was then filtered through gauze. After Triton X-100 was added to a final concentration of 1% (v/v), the filtrate was set on ice for 10 min. After centrifugation at 1,800 g for 10 min at 4°C, the pellet was kept and resuspended in 5 mL homogenization buffer. After centrifugation at 1,800 g for 8 min at 4°C, the pellet was kept and resuspended in 5 mL lysis buffer (10 mM pH 7.9 HEPES, 1.5 mM MgCl_2_, 10 mM KCl, 0.5 mM DTT, and 1.5 mM PMSF; PMSF and DTT were added prior to use). 2 M H_2_SO_4_ was added to the lysate drop-by-drop to a final concentration of 0.4 M. After incubation on ice for 1 hr, the mixture solution was centrifuged at 13,000 rpm for 10 min at 4°C. The supernatant was extracted and precipitated with trichloroacetic acid to a final concentration of 20%. After set on ice for 30 min, the samples were centrifuged at 13,000 rpm for 20 min at 4°C, and the pellet was washed with chilled acetone and dried overnight at 4°C. 

### 4.7. 2D Gel Electrophoresis

Histone-enriched proteins (150 *μ*g) were mixed with rehydration buffer (8 M Urea, 2% CHAPS, 20 mM DTT, 0.5% IPG buffer, and trace amount of bromophenol blue) to a final volume of 250 *μ*L and loaded onto 13 cm pH strips (pH 9–12). The strips were then rehydrated for 2 hr at 20°C, and first-dimension isoelectric focusing was performed by using the IPGphor IEF System (Amersham-Pharmacia Biotech): 20 V for 10 hr, 100 V for 1 hr, 500 V for 1 hr, 1,000 V for 1 hr, 2,500 V for 1 hr, and finally 8,000 V until the total V hr reached at least 80,000. Before second-dimension electrophoresis, the strips were equilibrated for 30 min with gentle shaking in SDS equilibration buffer (50 mM pH 8.0 Tris-HCl, 6 M urea, 3% (w/v) SDS, 20% (v/v) glycerol, and 0.125% (v/v) concentrated tributylphosphine). After equilibration, the strips were put on the top of 20% SDS-PAGE gels and sealed with agarose sealing solution (0.5% (w/v) agarose in SDS buffer plus a few grains of Bromphenol Blue). For western blot analysis, primary antibodies of streptavidin-HRP (Biosignaling) and core-histones (Abcam) were diluted 1 : 100 and 1 : 1,000, respectively. Secondary antibodies were diluted 1 : 5,000. Signal bands were detected with the Western Lightning chemiluminescence reagent (Perkin Elmer Inc.).

### 4.8. GST Pull-Down Assays

Purified GST-HCS1 proteins were first incubated with glutathione-sepharose beads for 1 hr at 4°C, and then 5 *μ*g His-Histone H3 proteins were added. After overnight incubation at 4°C, beads were washed extensively with assay buffer (10 mM Hepes, 100 mM NaCl, and 10 mM *β*-mercaptoethanol, pH 7.0) and resuspended in 2X SDS loading buffer. After boiling and centrifugation, the supernatant was separated by 15% SDS-PAGE. For western blot analysis, primary antibodies of GST and histone H3 (Santa Cruz Biotech) were diluted 1 : 1,000 and secondary antibodies were diluted 1 : 5,000. Signal bands were detected with the Western Lightning chemiluminescence reagent (Perkin Elmer Inc.).

## Figures and Tables

**Table 1:** Primers used for RT-PCR.

Construct name	Primer name	Primer sequence
Histone H3 (His-tag)	HistoneH3fw	CACCATGGCTCGTACCAA
	HistoneH3re	TTACAATCTTATGGTTCTAATCAAA

HCS1 (GST-tag)	HCSFW	GGGAATTCATGTTGTTTCTCATTTCTT
	HCSRE	GGCTCGAGTCATATTTTTCTTCGA
